# Editorial: New Insights Into Uveitis: Immunity, Genes, and Microbes

**DOI:** 10.3389/fimmu.2021.765377

**Published:** 2021-10-05

**Authors:** Peizeng Yang, Shigeaki Ohno, Manfred Zierhut

**Affiliations:** ^1^ The First Affiliated Hospital of Chongqing Medical University, Chongqing, China; ^2^ Department of Ophthalmology, Faculty of Medicine and Graduate School of Medicine, Hokkaido University, Sapporo, Japan; ^3^ Centre for Ophthalmology, University of Tuebingen, Tuebingen, Germany

**Keywords:** uveitis, immunity, genes, microbes, pathogenesis

Uveitis, comprised of a clinically heterogenous group of more than 100 subtypes with diverse etiologies, is one of the leading causes of blindness worldwide. Both innate and adaptive immune responses are reported to be actively involved in the development of uveitis. The occurrence of uveitis is multifactorial, with genetic inheritance, immune dysregulation, gut microbiota abnormalities, and environmental factors involved. This Research Topic aimed at bringing together contributions covering various aspects related to the pathogenesis of uveitis, with the hope to drive forward a more detailed and in-depth understanding of this challenging disease.

Genetic background is an important predisposing factor for uveitis. Takeuchi et al. contribute a comprehensive review to summarize recent findings regarding the genetic pathogenesis of non-infectious uveitis. Multiple MHC and non-MHC genes have been identified to participate in the pathogenesis of various uveitis subtypes. They indicate that the Th17 immune response is a common key in the pathogenesis of non-infectious uveitis. With growing understanding of the predisposing genetic background, a personalized and precise treatment strategy based on the patient’s genetic make-up could be expected in the future. Pei et al. studied the association of SNP polymorphisms of the IL33/ST2 gene with Behcet’s disease. They report that rs3821204 is associated with the development of this disease, and the frequency of rs2210463 G allele is lower in patients with genital involvement. Kuiper and Venema review the relationship between the HLA-A29 serotype and Birdshot Uveitis. They discuss how key amino acid positions of HLA-A29 impact the peptide binding preference and interaction with T cells and to what extent the risk genes ERAP1 and ERAP2 affect HLA-A29. They also argue why Birdshot Uveitis only affects HLA-A29-positive individuals. Huang et al. studied the association of SNP polymorphisms in CTLA-4 and PD-1 genes with Posner-Schlossman Syndrome (PSS) in a southern Chinese population. They report that the frequencies of the CACGG haplotype (rs733618-rs4553808-rs5742909-rs231775-rs3087243) of CTLA-4 and the TGAGC haplotype (rs10204525-rs2227981-rs2227982-rs41386349-rs36084323) of PD-1 in the PSS group are significantly lower than those in the control group. Circulating plasma levels of sCTLA-4 and sPD-1 in PSS patients are significantly higher than those in controls. Yang et al. address the SNP polymorphisms of complement genes in PSS patients. Rs800292 at the CFH gene is significantly associated and the additive effect of CFH-rs800292 and SERPING1-rs3824988 is identified. Furthermore, the rs800292 AA genotype is associated with a higher intraocular pressure and higher frequency of recurrence.

Multiple articles in this Research Topic focus on the imbalanced immune system. Wildner and Diedrichs-Möhring contribute a mini review to introduce the concept of “molecular mimicry” and describe a variety of non-ocular antigens that mimic retinal autoantigens. T cells that are activated by mimotopes outside of the eye can pass the blood-retina barrier and enter ocular tissues and when reactivated by cross-reaction with autoantigens in the eye, they induce uveitis by recruiting inflammatory cells. Xu et al. investigated the aqueous metabolic profiles in Vogt-Koyanagi-Harada (VKH) and Behcet’s disease (BD). Twenty-eight and 29 differential metabolites are identified in the VKH and the BD groups compared with a control group respectively. Pathway enrichment analysis illustrates pantothenate and CoA biosynthesis are altered when comparing VKH with the control group, while D-arginine, D-ornithine and phenylalanine metabolism are altered when comparing BD with the control group. Aminoacyl-tRNA biosynthesis is altered in both VKH and BD groups when compared to controls. Matas et al. conducted a multicenter, prospective, observational, 12-month follow-up study of 60 patients with uveitic macular edema (UME). They report that increased levels of Tregs and reduced levels of IL-6 in serum may be prognostic factors of sustained anatomical improvement in UME. Ko et al. illustrate that CD73^+^ dendritic cells (DCs) trigger cascading Th17 responses, and the activated Th17 cells expressing CD73 could further augment Th17 responses, leading to cascading exacerbation. The findings indicate that disabling the CD73 function of DCs might help to block this cascading response and mitigate Th17 responses. Barfüβer et al. investigated the altered metabolic phenotype of immune cells in a spontaneous autoimmune uveitis model, the “Equine Recurrent Uveitis (ERU) model”, revealing that PBMCs in ERU show a more active metabolic phenotype in basal state by upregulating both the oxidative phosphorylation and the glycolytic pathway. They also report an increased compensatory glycolytic rate of PBMCs and CD4^+^ T cells of ERU cases under mitochondrial stress conditions. Degroote and Deeg also focus on ERU, which is with strong clinical and pathological resemblance to autoimmune uveitis in human. They review latest studies regarding the pathogenesis-associated processes of ERU, from perspectives of both cellular and molecular levels. Bansal and Gupta draw attention to the importance of biomarker identification in multiple uveitis subtypes, which usually manifest as nonspecific or atypical clinical presentation. Proteomics is a high throughput technology and a powerful screening tool for biomarkers. They review recent studies using proteome analysis to identify biomarkers in different ocular fluids (vitreous, aqueous, or tears) from various types of uveitis. Egwuagu et al. summarize the advances in molecular cell biology and immunology over the past 30 years that have contributed to the understanding of immunopathogenesis of uveitis and particularly emphasize on how advances in biotechnology have facilitated the development of biologics and cell-based immunotherapies for uveitis and other neuroinflammatory diseases. Bradley et al. in their review discuss the importance of quantitative assessment of experimental ocular inflammatory disease. They highlight three linked but distinct clinical states in EAU (experimental autoimmune uveitis) model that produce retinal vulnerability to inflammation. Deploying longitudinal, multimodal imaging approaches can greatly help the analysis of tissue changes in architecture, cell content and function. Jiang et al. analyzed the systemic response of diverse immune cells to glucocorticoids (GC) therapy in VKH syndrome. Advanced activation and differentiation, as well as dysregulated numbers of peripheral lymphocytes are the major immunological features of VKH. GC therapy with methylprednisolone not only inhibits T cell activation, but also affects monocyte subsets, which might combinatorically lead to the inhibition of the pathogenic immune response.

Evidence is accumulating in favor of an important role of microbiota in uveitis pathogenesis. Li et al. bring the term “ocular microbiota” to our attention since a microbial etiology is indicated in various inflammatory eye diseases. They contribute an overview of the literature on ocular microbiota and the role of commensal microbes in several eye diseases, discussing the interaction between microbial pathogens and host factors, and evaluating therapeutic potential of targeting ocular microbiota to treat intraocular inflammation. Ryan et al. investigated the pathogenesis of Zika-associated anterior uveitis using an *in vitro* human cell model. Interactions between ZIKA and human iris pigment epithelial cells were investigated with infectivity assays and RNA-sequencing. The results suggest that iris pigment epithelium mounts a molecular response that limits intraocular pathology in most individuals.

Predisposing factors of uveitis do not work alone. Instead, a widely accepted hypothesis is that an infectious agent and immunological abnormalities in genetically susceptible individuals may be responsible for the initiation and chronicity of uveitis. Mehmood et al. contribute a review to describe the relationship between polymorphisms in genes involved in gut biology and changes in the microbiome of Behcet’s patients. A potential decrease in bacterial species producing short chain fatty acids links to mutations in genes involved in their production. Mölzer et al. focus on immune privilege of the eye, which can be breached and the eye is still susceptible to intraocular inflammation. They contribute a comprehensive review discussing the pathogenesis of uveitis in the context of immune privilege, infection, environment, and microbiome. Wakefield et al. summarize recent developments regarding the pathogenesis, clinical features, and effective treatment of acute anterior uveitis. Accumulating evidence confirmed anterior uveitis as a polygenic disease, and the interaction between HLA-B27 and gut microbiome is indicated in experimental animals. They introduce the typical features of acute anterior uveitis and its response to topical, regional and systemic immunosuppressive treatment and highlight anti-cytokine therapy (anti-TNF and anti-IL-6) in severe and recurrent cases. Using the “HSV-1 infected mouse Behçet’s disease model”, Islam et al. demonstrate that environment and stress (including anxiety stress, cold stress, oxidative stress and noise stress) influence the incidence of disease. Microbial diversity due to environmental differences might be one explanation for regional differences in the incidence of Behcet’s disease.

We would like to extend our sincere gratitude to all the authors who have participated in this Research Topic. Their contributions will surely increase our understanding of the pathogenesis of uveitis, which is of great significance to facilitate an early and correct diagnosis of disease and also to the development of more effective therapeutic approaches.

## Author Contributions

All authors contributed to the article and approved the submitted version.

## Funding

National Natural Science Foundation Key Program (81930023), Natural Science Foundation Major International (Regional) Joint Research Project (81720108009), Chongqing Outstanding Scientists Project (2019), Chongqing Key Laboratory of Ophthalmology (CSTC, 2008CA5003), Chongqing Science & Technology Platform and Base Construction Program (cstc2014pt-sy10002) and the Chongqing Chief Medical Scientist Project (2018).

## Conflict of Interest

The authors declare that the research was conducted in the absence of any commercial or financial relationships that could be construed as a potential conflict of interest.

## Publisher’s Note

All claims expressed in this article are solely those of the authors and do not necessarily represent those of their affiliated organizations, or those of the publisher, the editors and the reviewers. Any product that may be evaluated in this article, or claim that may be made by its manufacturer, is not guaranteed or endorsed by the publisher.

